# B-cells Drive Response to PD-1 Blockade in Glioblastoma Upon Neutralization of TGFβ-mediated Immunosuppression

**DOI:** 10.21203/rs.3.rs-2399170/v1

**Published:** 2023-01-09

**Authors:** David Hou, Brandyn Castro, Mark Dapash, Andrew Zolp, Joshua Katz, Víctor Arrieta, Jana Biermann, Johannes Melms, Jan Kueckelhaus, Jasim Benotmane, Mark Youngblood, Aida Rashidi, Leah Billingham, Crismita Dmello, Gustavo Vazquez-Cervantes, Aurora Lopez-Rosas, Yu Han, Ronit Patel, Tzu-yi Chia, Lu Sun, Robert Prins, Benjamin Izar, Deiter Henrik Heiland, Peng Zhang, Adam Sonabend, Jason Miska, Maciej Lesniak, Junfei Zhao, Catalina Lee-Chang

**Affiliations:** Northwestern University; Northwestern University; Northwestern University; Northwestern University; Northwestern University; Northwestern University; Columbia University; Columbia University Irving Medical Center; University of Freiburg; University of Freiburg; Northwestern University; Northwestern University; Northwestern University; Northwestern University; Northwestern University; Northwestern University; Northwestern University; Northwestern University; Northwestern University; UCLA; University of California, Los Angeles; Columbia University Irving Medical Center; University of Freiburg; Northwestern University; Northwestern University; University of Miami Miller School of Medicine; Northwestern University; Columbia University; Northwestern University

## Abstract

Immunotherapy has revolutionized cancer treatment but has yet to be translated into brain tumors. Studies in other solid tumors suggest a central role of B-cell immunity in driving immune-checkpoint-blockade efficacy. Using single-cell and single-nuclei transcriptomics of human glioblastoma and melanoma brain metastasis, we found that tumor-associated B-cells have high expression of checkpoint molecules, known to block B-cell-receptor downstream effector function such as plasmablast differentiation and antigen-presentation. We also identified TGFβ-1/TGFβ receptor-2 interaction as a crucial modulator of B-cell suppression. Treatment of glioblastoma patients with pembrolizumab induced expression of B-cell checkpoint molecules and TGFβ-receptor-2. Abrogation of TGFβ using different conditional knockouts expanded germinal-center-like intratumoral B-cells, enhancing immune-checkpoint-blockade efficacy. Finally, blocking αVβ8 integrin (which controls the release of active TGFβ) and PD-1 significantly increased B-cell-dependent animal survival and immunological memory. Our study highlights the importance of intratumoral B-cell immunity and a remodeled approach to boost the effects of immunotherapy against brain tumors.

## Introduction

Glioblastoma (GBM) is an immunologically cold tumor characterized by minimal lymphocyte infiltration and significant myeloid cell prevalence. Besides the intrinsically low mutational burden of GBM^1^, the suppressive tumor microenvironment (TME) plays a major role in developing a noninflamed tumor^2,3^. Key drivers of this immunosuppression include a combination of secreted soluble factors such as cytokines and prostaglandins. Immunosuppression is also mediated by cellular components such as regulatory T-cells (Tregs), regulatory B-cells (Bregs), myeloid-derived suppressor cells (MDSCs), and tumor-associated macrophages (TAMs) ^3,4^. Together through pathways such as the programmed cell death protein/programmed death ligand 1 (PD-1/PDL-1) axis or CD155 (poliovirus receptor) pathway, these players work together to inhibit effector functions of immune cells ^5,6,7^.

While immune checkpoint blockade therapy has revolutionized the treatment of many solid cancers, these results have yet to be translated to GBM^8^. In contrast to the lymphocyte-excluded nature of GBM, melanoma brain metastases (MBM) are characterized by an increase in lymphocyte infiltration and a decrease in myeloid cells with clinically meaningful intracranial efficacy for immune checkpoint blockade ^9,10^. However, patients with MBMs exhibit worse response to checkpoint blockade compared to patients with metastatic melanoma that does not involved the central nervous system^11^. Thus, melanoma brain and extracranial metastases serve as a control tumor model to study both the larger CNS role and tumor-intrinsic properties in GBM immune suppression.

B-cells are an understudied yet critical player in brain tumor immunity that can serve both regulatory and anti-tumor functions^12,13^. B-cells begin at a naïve state and upon BCR and cytokine stimulation, undergo differentiation into non-switched IgM^+^ memory B-cells within the germinal center ^14^. Further activation leads to isotype switching and development into switched memory B-cells, which can then mature into plasmablasts and plasma cells-the most activated B-cell state ^14^. The function of antitumoral B-cells involved in immune checkpoint blockade efficacy include differentiation into plasmablast^15^ and the subsequent production of tumor-reactive antibodies ^16,17,18,19^, as well as T-cell activation via antigen-presentation^20,21^ or complement ^22^.

On the other hand, immune suppressive B-cells can undermine anti-tumor immunity. We previously showed that B-cells infiltrating the GBM could be converted into immunosuppressive regulatory B-cells by local myeloid cells, inhibiting T-cell function^12^. B-cells also express inhibitory checkpoint molecules that drive their functional suppression via immunoreceptor tyrosine-based inhibitory motifs (ITIMS)^23^. Several inhibitory checkpoint molecules have been well studied in B-cells, including CD72, FcyR2 (CD32), and CD22, among others ^23,24,25,26,27^. The expression kinetics of B-cell checkpoint molecules differ from those of T-cell checkpoint molecules in that naïve B-cells express elevated levels of checkpoint molecules to prevent tonic B-cell Receptor (BCR) signaling, and downregulation of checkpoint molecules is required for differentiation of B-cells into plasmablasts ^28,29^. B-cells are also susceptible to many cytokines that can play a regulatory role in B-cell function, such as IL10, IL4, IL7, and the tumor growth factor beta (TGFβ) superfamily, including bone morphogenic progenitor (BMP) ^30–32 31 33–35 36 33^. Studies have also linked B-cell stimulation by these cytokines with elevated expression of checkpoint molecules ^32^.

New high-resolution techniques such as spatial transcriptomic, immunofluorescence multiplex analysis, and single-cell and nuclei transcriptomic sequencing allow us to better characterize the interactions of B-cells in GBM. The role of B-cells in cancer development and immunological treatment has gained renewed interest, as numerous studies have shown that tumor-infiltrating B-cells have a positive prognostic value in other solid tumors such as breast cancer^37^, colorectal cancer ^38,39^, non-small cell lung cancer ^40^, head and neck cancer ^41^, ovarian cancer ^42^, biliary tract cancer^43^, primary cutaneous melanoma^15,44^, metastatic melanoma^45^, and hepatocellular carcinoma ^46^. Studies have also highlighted that intratumoral B-cell infiltration and formation of tertiary lymphoid structures (TLS) may play a significant role in immune checkpoint blockade efficacy^47,48,49^. In advanced metastatic melanoma patients, TLS and B-cell signature (but not T-cells) predicted therapeutic response to pembrolizumab and ipilimumab (anti-CTLA-4)^45^. Therefore, a better understanding of the B-cell landscape can provide insight into improving the efficacy of existing immunotherapies for GBM and developing novel treatments.

In this study, given the diverse phenotype and role that B-cells play in cancer, we characterized B-cell spatial profile, single-cell transcriptome, and immune phenotype of intratumoral B-cells in GBM, MBM, and extracranial melanoma metastases (ECM). We demonstrated how brain tumor TMEs negatively regulate B-cell function and explored the role of targeting B-cell suppression to enhance checkpoint blockade efficacy and promote anti-tumor immunity.

## Results

### B-cell Localization and Activation Status in Brain Tumors

**Figure 1:**
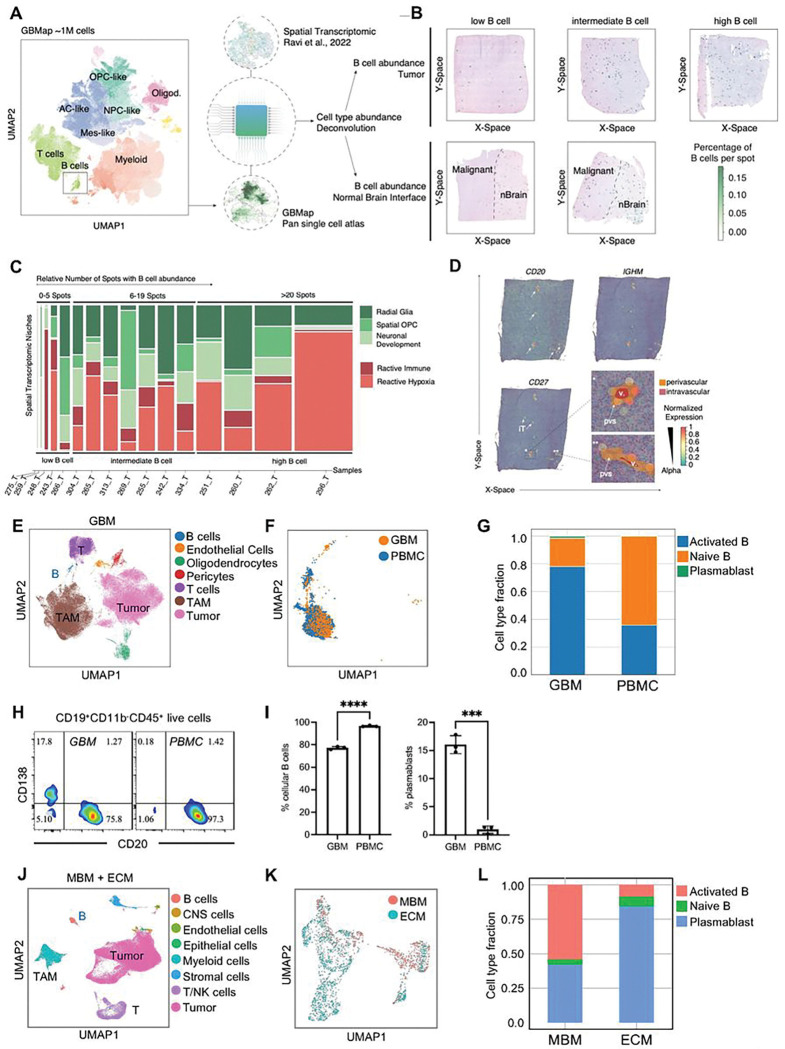
B-cell Location and Activation Status in Brain Tumors **(A)** Illustration of the workflow for integrating spatial transcriptomic and the reference GBMap dataset to infer B-cell abundance in the tumor and normal brain interface regions. **(B)** Representative surface plots of B-cell accumulation per spatial transcriptomic spot using the RCDT deconvolution algorithm. On top, representative samples of low-, intermediate and high B-cell infiltration. **(C)** Mosaic plot of B-cell abundance across different transcriptional niches. **(D)** Surface plot of B-cell marker expression and intratumoral, per- or intravascular accumulation of B-cells. Arrows mark areas of B-cell infiltration. **(E)** UMAP plot of GBM samples enriched on CD45 pooled from the UCLA dataset (n=40), GSE182109 dataset (n=22), and Northwestern dataset (n=2) showing cell type assignments. **(F)** UMAP plot of B-cells from GBM samples and paired PBMC samples (UCLA dataset n=13, Northwestern dataset n=2) showing cell origin. **(G)** Activation status distribution of B-cells from GBM samples and paired PBMC samples analyzed from single-cell RNA sequencing data. **(H)** Representative flow cytometry plot showing the plasmablast population (CD19+CD138+CD20−) in GBM B-cells compared to PBMC B-cells. **(I)** Bar graphs analyzing GBM and PBMC samples showing percent B-cells out of total lymphocytes as well as percent plasmablast within total B as determined via flow cytometry. **(J)** UMAP plot of 17 melanoma brain metastases and 10 extracranial metastases showing cell type assignments. **(K)**UMAP plot of B and plasma cells from 15 MBM and 8 ECM samples depicting the tissue of origin. **(L)** Activation status distribution of B-cells from MBM and paired ECM samples analyzed from single nuclei RNA sequencing data. ns= p>0.05, * = p<0.05, ** = p<0.01, *** = p<0.001, **** = p<0.0001

First, we characterized the abundance and localization of intratumoral B-cells in GBM using single-cell deconvolution of spatially resolved transcriptomic profiling and B-cell marker expression (*CD20*, *IGHM*, and *CD27*) of 16 human GBM specimens (Fig. 1A and B). Distribution and accumulation across all samples revealed high heterogeneity, including higher B-cell abundance (> 20 B-cell-enriched spots, n = 4), intermediate (5–19 B-cell-enriched spots, n = 7), and low B-cell abundance (0–4 B-cell-enriched spots n = 5) (Fig. 1B and C). However, not all tumors had infiltration of B-cells. This was evidenced by samples that had robust B-cell accumulation in benign peritumoral regions but minimal B-cells infiltration within the tumor mass (Fig. 1B). Thus, the spatial analysis excluded those B-cells located in the peritumoral brain tissue. Study of co-localization demonstrated an absence of explicit assignment to transcriptional niches in contrast to myeloid or T-cell accumulation as described recently ^50^ (Fig. 1C). Moreover, B-cells could be characterized relative to different zones of the GBM tumor, including regions with abundant radial glia, oligodendrocyte progenitor cells (OPC), neuronal development, reactive immune response, and reactive hypoxia. We grouped tumor samples based on levels of B-cell infiltration, and in tumors with high infiltration, B-cells tended to cluster within zones of reactive immune response and hypoxia (Fig. 1D). Further spatial analysis of intratumoral B-cell accumulation revealed three patterns encompassing tissue-resident B-cell infiltration, and perivascular and intravascular B-cell accumulation. The most common pattern was a perivascular accumulation, further confirmed by spatial weighted correlation analysis in which perivascular space was highly significantly co-localized (Sup Fig. 3B, vascular analysis).

To gain an understanding of the functional status of B-cells in brain tumors, we studied their activation status in both GBM and MBM tissues. Using single-cell RNA sequencing, we analyzed the TME of primary GBM tumor tissues pooled across multiple patient cohorts and identified the B-cell population (Fig. 1E). From the pooled GBM tumor samples, we had 15 paired peripheral blood mononuclear cells (PBMC) single-cell data. Tumoral B-cells and B-cells from patient PBMCs were stratified based on B-cell subsets, including activated B cells, naïve B cells, and plasmablasts. The data show that most tumoral B-cells had undergone activation compared to PBMC B-cells (Fig. 1F–G). Flow cytometry analysis of a GBM tumor sample and paired PBMCs for B-cell infiltration and plasmablast phenotype showed decreased cellular B-cells in the tumor (CD20^+^ B-cells), but an increased proportion of plasmablasts (CD20^−^CD138^+^ B-cells) compared to PBMC B-cells (Fig. 1H–I).

For a non-GBM CNS cancer control, we also analyzed the TME of both MBM and extracranial tumors (ECM), identifying B-cells in both brain metastasis and extracranial samples (Fig. 1J–K). Upon stratifying B-cells into naïve, activated, and plasmablast subsets, we found that B-cells in extracranial melanoma tumors were over 75% plasmablasts. In contrast, MBM B-cells mainly consisted of activated B-cells with less than 50% plasmablasts (Fig. 1L). Our data suggest that tumor-infiltrating B-cells across GBM and MBM are antigen-experienced, with GBM having significantly reduced plasmablasts than both MBM and ECM. However, MBM have fewer plasmablasts compared to ECM.

Building from our observations in human GBM and MBM, we determined that similar B-cell phenotype exists in our murine CT2A orthotopic glioma models. Nur77-GFP mice (where lymphocytes express GFP upon receptor activation via antigen crosslink ^51^) were injected with CT2A glioma cells and allowed to develop tumors. We found that B-cells in the tumor expressed elevated GFP compared to B-cells in the periphery (deep cervical lymph node), suggesting that tumor B-cells had undergone B-cell receptor (BCR) activation (Sup Fig. 1A).

### Checkpoint Molecule Expression in Tumor B-cells

**Figure 2:**
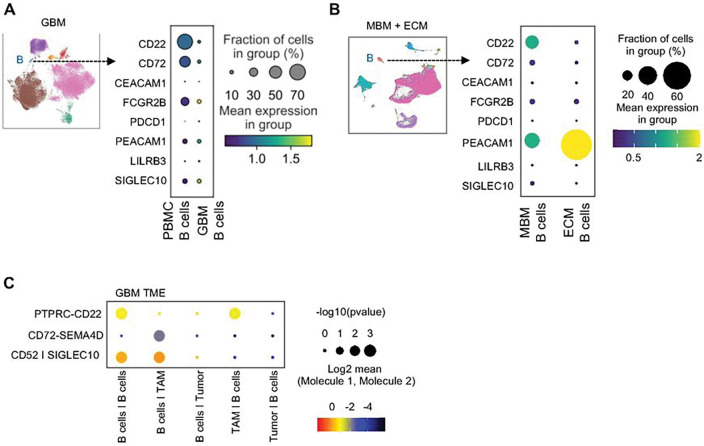
B-cells in brain tumors express inhibitory checkpoint molecules **(A)** Dot plot of inhibitory checkpoint molecule expression from GBM B-cells and PBMC B-cells analyzed from single-cell RNA sequencing. Increasing dot size correlates to increased prevalence within the entire B-cell population, increasing yellow intensity correlates to increased checkpoint molecule expression per B-cell. **(B)** Dot plot of inhibitory checkpoint molecule expression from MBM compared to ECM. Increasing dot size correlates to increased prevalence within the entire B/plasma cell population, increasing yellow intensity correlates to increased checkpoint molecule expression across B/plasma cell populations. **(C)**Representative flow cytometry plot showing CD22 and FCGR2B expression on GBM plasmablasts and bar plot showing CD72, CD22, and FCGR2B expression on GBM plasmablasts. **(D)** Bar plots showing expression of CD72, CD22, and FCGR2B on total B-cells in GBM tumors and PBMC. **(E)** Dot plot of an unbiased interactome analysis between B-cells and B-cells, TAMs, or tumor cells showing potential interactions between CD22 on B-cells and PTPRC on B-cells and TAMs, CD72 on B-cells and SEMA4D on TAMs, and SIGLEC10 on B-cells and CD52 on other B-cells are upregulated. The complete unbiased interactome analysis is shown in supplemental figure 2. ns= p>0.05, * = p<0.05, ** = p<0.01, *** = p<0.001, **** = p<0.0001

Under physiologic conditions, naïve B-cells express checkpoint molecules that suppress BCR signaling and prevent autoimmunity via their intracellular ITIM motifs ^52^. Known B-cell checkpoint molecules include CD22, CD72, CEACAM1, FCGR2B, PDCD1, PECAM1, LILRB3, and SIGLEC10 ^24,53,54,55,56,57^. Contrasting the kinetics of T-cells where activation increases checkpoint molecule expression ^58,59^, studies have shown that significant downregulation of CD22 and CD72 is required to mature B-cells into a plasmablast state ^28,29^. Compared to PBMC B-cells, GBM tumoral B-cells had decreased expression of CD22, CD72 (Fig. 2A). However, comparison of checkpoint molecule expression on tumor B-cells relative to other checkpoint molecules on the same cells show tumor B-cells still maintain elevated expression of CD22, CD72, and FCGR2B (Sup Fig. 1C). In melanoma samples, we saw higher levels of *CD22* in intratumoral B-cells of MBM than in ECM B-cells (Fig. 2B).

Using single-cell transcriptomic data, we found that B-cells in murine CT2A tumors also express elevated *Cd72, Fcgr2b*, and *Cd22* (Sup Fig. 1D-E). These checkpoint molecules, as well as downstream ITIM signaling molecules, SHIP1 and SHIP2 ^60^, were upregulated in the tumor B-cells compared to splenic B-cells at both the transcription and protein levels (Sup Fig. 1D-E).

We then conducted an unbiased interactome analysis in GBM and murine CT2A tumors using single-cell transcriptomic data to rank B-cell surface molecules and paired receptors or ligands on other cells based on expression levels (Sup Fig. 2A-B). From this analysis, we found elevated expression of *CD22, CD72*, and *SIGLEC10* paired with elevated expression of their respective ligands *PTPRC, SEMA4D*, and *CD52* on other B-cells, TAMs, or tumor cells (Fig. 2C).

Our results suggest that B-cells in human GBM and melanoma brain metastases, as well as murine CT2A glioma models, are activated but express elevated levels of key checkpoint molecules such as CD22/Cd22 and CD72/Cd72, leading to an inability to differentiate into plasmablasts fully.

#### Brain Tumors Produce B-cell-suppressing Cytokines with Corresponding Receptors Expressed by Intratumoral B-cells.

Having established that B-cells in GBM, MBM, and murine CT2A models are unable to fully differentiate into plasmablasts and have elevated expression of B-cell checkpoint molecules, we then sought to identify factors produced by the tumor and its TME that prevented B-cells from becoming fully activated and entering a plasmablast state.

**Figure 3:**
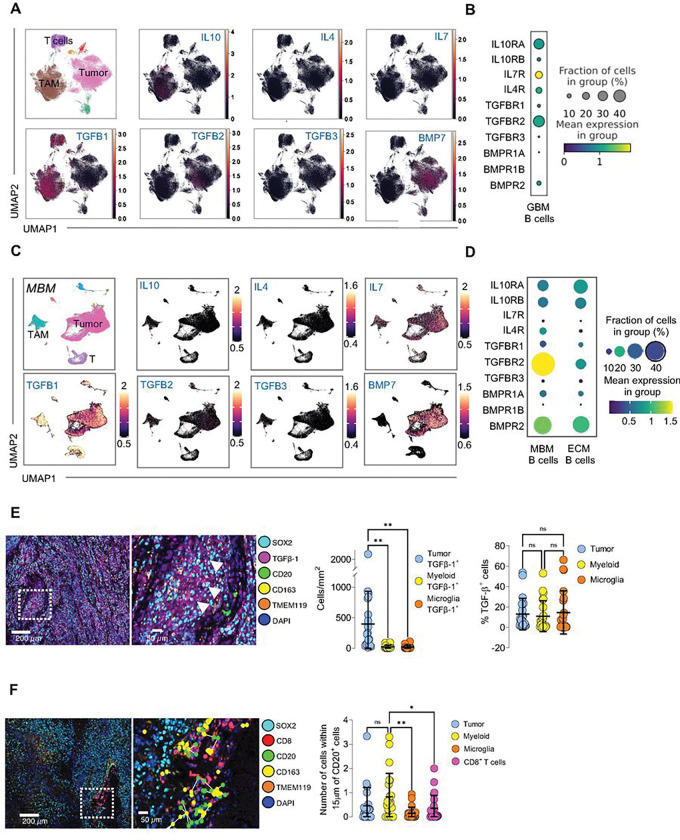
GBM and MBM produce soluble B-cell suppressive factors **(A)** UMAP plots of the GBM tumor environment showing RNA expression of B-cell suppressive cytokines *IL10, IL4, IL7, TGFB1, TGFB2, TGFB3*, and *BMP7*. *TGFB1* is highly expressed in the GBM TME by myeloid cells. *BMP7*, also in the TGF beta super family, is expressed by tumor cells. **(B)** Dot plot of suppressive cytokine receptor expression on intratumoral GBM B-cells. Intratumoral B-cells express *TGFBR2*. **(C)** UMAP plots of melanoma metastases tumor environment showing RNA expression of B-cell suppressive cytokines *IL10, IL4, IL7, TGFB1, TGFB2, TGFB3, and BMP7*. *TGFB1* is highly expressed in the melanoma metastases TME by TAMs, T-cells, and tumor cells. *BMP7* and *IL7* are also expressed by tumor cells. **(D)** Dot plot of suppressive cytokine receptor expression on MBM and ECM B-cells. Intracranial B-cells have increased expression of *TGFBR2* compared to extracranial B-cells. **(E)** Spatial multiplex immunofluorescence analyzing cellular infiltration and TGFb-1 expression in the GBM tumor microenvironment (n=21). The TME was analyzed for prevalence of TGFb-1^+^ tumor cells (SOX2^+^), myeloid cells (CD163^+^TMEM119^−^), and microglia (TMEM119^+^CD163^−^), as well as TGFb-1 expression within the cell populations. TGFb-1^+^ cells in the TME mainly consist of tumor cells. **(F)** Spatial multiplex immunofluorescence analyzing distance between B-cells and other cell types in the GBM TME (n=21). Cells within 15 mm of B-cells were quantified to predict possible B-cell interactions. Myeloid cells tend to be the most common cell type within 15 mm of B-cells. ns= p>0.05, * = p<0.05, ** = p<0.01, *** = p<0.001, **** = p<0.0001

Several known cytokines and pathways inhibit B-cell function, such as IL10, IL7, IL4, and the TGFβ superfamily ^32^. Using our single-cell transcriptomic data for the GBM TME, we analyzed the expression of suppressive cytokines based on cell type clustering. We found that TGFβ-1 was highly expressed by T-cells and tumor-associated macrophages (TAMs) (Fig. 3A, Sup Fig. 3A). BMP7, which is part of the TGFβ superfamily, was also found to be expressed by tumor cells. Next, we analyzed B-cells expressing paired cytokine receptors and found that *TGFBR2* was elevated (Fig. 3B). The three different TGFβ-R forms (TGFβ-R1, TGFβ-R2, and TGFβ-R3) can form both homo- and hetero-dimers, which allows TGFβ−1 signaling to occur through TGFβ-R2 ^61^. While single-cell transcriptomic data pointed to myeloid cells as the primary producers of TGFβ−1 in the tumor environment, spatial transcriptomics highlighted myeloid cells as correlated most with B-cells for spatial interactions (Sup Fig. 3B).

Because B-cells in MBM exhibited similar functional suppression as observed in GBM, we also analyzed TME suppressive cytokine expression and intratumoral B-cell cytokine receptor expression using single-nuclei transcriptomic data. As we observed in GBM, the TME in MBM also expresses elevated levels of *TGFB1* associated most strongly with myeloid cells (Fig. 3C, Sup Fig. 3C). Inhibitory cytokine receptor analysis on intracranial B-cells showed the highest expression of *TGFBR2* compared to other receptors and elevated *TGFBR2* on intracranial B-cells compared to extracranial B-cells (Fig. 3D).

We ran the same analysis in our murine CT2A model and found *Tgfb1* and *Bmp7* expressed by myeloid and tumor cells, respectively (Sup Fig. 3D). *Tgfb3* was also expressed by tumor cells. Comparison between brain tumor and peripheral myeloid cells showed that expression of TGFβ−1 on myeloid cells was unique to tumor-resident myeloid cells (Sup Fig. 3E). Receptor analysis on intratumoral B-cells also showed high levels of *Tgfbr2* and *Tgfbr1* (Sup Fig. 3F), and *Tgfbr2* was elevated on intratumoral B-cell compared to splenic B-cells within the same animal (Sup Fig. 3G-H). GSEA enrichment analysis showed significantly increased expression of TGFβ responsiveness genes in B-cells in the tumor compared to B-cells in the spleen (Sup Fig. 3I).

Next, we used spatial multiplex immunofluorescence to analyze TGFβ−1 expression based on cell type and distance from B-cells. We analyzed human GBM sections for tumor cells (SOX2^+^), myeloid cells (CD163^+^TMEM119^−^), microglia (TMEM119^+^CD163^−^), T-cells (CD8^+^), B-cells (CD20^+^), and TGFβ−1. TGFβ−1^+^ cells in the TME consisted mainly of SOX2^+^ tumor cells, but the proportion of TGFβ−1^+^ within each cell population was similar between tumor cells, myeloid cells and microglia despite lower cell counts for the latter two cell types (Fig. 3E). We also analyzed the distance between B-cells and other cell types since TGFβ activation and signaling require proximity between recipient B-cells and the TGFβ source ^62^. We characterized the different cell types within a 15 μm radius of B-cells, as previous studies had demonstrated that 15 μm is a close distance enough to both form an immune synapse and initiate TGFβ−1 release ^62,63^, and found that myeloid cells were the most common cell type within 15 μm radius of B-cells (Fig. 3F).

Our data suggest that tumor and myeloid cells within the GBM TME produce of suppressive cytokines, particularly TGFβ−1, that may interact with B-cells and impact their maturation and function.

### TGFβ Inhibits B-cell Differentiation and Induces Checkpoint Molecule Expression

**Figure 4:**
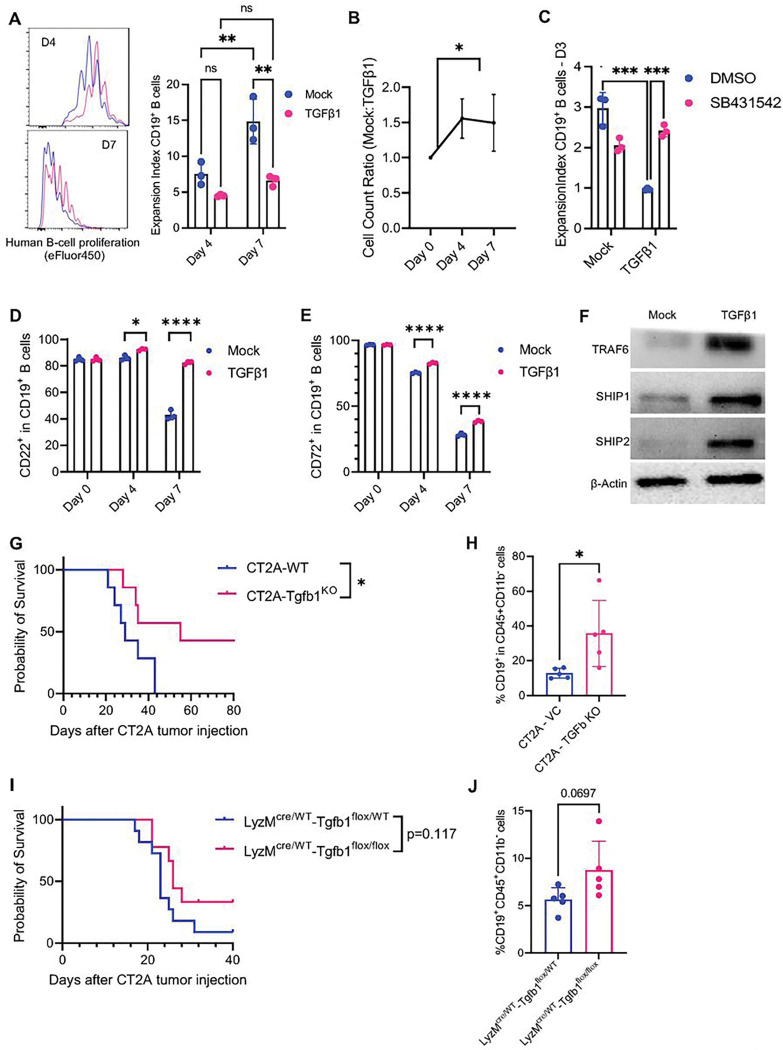
TGFβ−1 Inhibits B-cell proliferation and maintains B-cell checkpoint molecule expression. **(A)** Histogram and bar plots showing cellular expansion of activated B-cells cultured with exogenous TGFb-1or mock as determined by efluor-450 proliferation dye. B-cells were activated with a B-cell expansion cocktail and TGFb-1 was added every day starting from day 0. Addition of TGFb-1inhibited B-cell proliferation. **(B)** B-cell proliferation was also quantified based on cell counts. The same number of cells were plated in each group, and the ratio of mock B-cells to TGFb-1 B-cells was determined at days 4 and 7. **(C)** Bar plot of B-cell expansion showing TGFb-1 induced loss of proliferation could be prevented with a TGFβ-R inhibitor. B-cells were activated with an expansion cocktail and cultured with exogenous TGFβ−1. SB431542 small molecule TGFβ-R inhibitor was also added every day to the experimental group 30 minutes before addition of TGFβ−1. While TGFβ−1 inhibited B-cell proliferation, blockade of the TGFβ receptor was able to maintain normal B-cell proliferation even in the presence of exogenous TGFβ−1. **(D-E)** Bar plots showing CD22 and CD72 expression on B-cells after culture with TGFβ−1. B-cells were activated with B-cell expansion cocktail and TGFβ−1 was added every day starting from day 0 in the experimental group. While control B-cells decreased expression of CD22 and CD72 as they became more activated, additional of TGFβ−1 maintained elevated levels of CD22 and CD72. **(F)** Western blot for TRAF6, SHIP1, and SHIP2 in B-cells cultured for 4 days with or without TGFβ−1. Culture with TGFβ−1 led to an increase in TRAF6, SHIP1, and SHIP2 in B-cells. **(G)** Lyz2-Tgfb1 transgenic mice with *Tgfb1* knocked out of myeloid cells (LyzM^cre/WT^-Tgfb1^flox/flox^) were challenged with CT2A glioma cells. Animal survival was assessed in these experimental mice compared to wild-type controls (LyzM^cre/WT^-Tgfb1^flox/WT^). Knockout of *Tgfb1* trended towards improved animal survival. **(H)** Lyz2-Tgfb1 transgenic mice with *Tgfb1* knocked out of myeloid cells (LyzM^cre/WT^-Tgfb1^flox/flox^) were challenged with CT2A glioma cells. After 14 days, the tumors were analyzed for B-cell infiltration via flow cytometry. Knockout of *Tgfb1* trended towards increased B-cell counts in the tumor. **(I)** Wild-type C57 mice were challenged with CT2A glioma cells with *Tgfb1* knocked out (CT2A-Tgfb1^KO^). Animal survival was assessed in these experimental tumors compared to wild type tumors (CT2A-Tgfb1^VC^). Knockout of *Tgfb1* in CT2A tumor cells significantly improved animal survival. **(J)** The tumors of wild-type mice bearing CT2A-Tgfb1^KO^ tumors were analyzed after 14 days for B-cell infiltration compared to mice bearing CT2A-Tgfb1^VC^ controls. Knockout of *Tgfb1* in CT2A tumor cells significantly increased B-cell counts in the tumor. ns= p>0.05, * = p<0.05, ** = p<0.01, *** = p<0.001, **** = p<0.0001

To determine the direct effects of TGFβ signaling on B-cells, we cultured human B-cells from healthy donors in a B-cell expansion media supplemented with or without exogenous TGFβ. We found that the B-cell expansion media could expand naïve B-cells. TGFβ−1 significantly inhibited B-cell expansion as measured by cell proliferation and cell counts (expansion index mean±SD: Day 7 B-cell + DMSO vs. Day 7 B-cell + TGFβ−1 14.83 ± 2.53 vs 6.60 ± 0.59, p < 0.01, Fig. 4A–B). This inhibitory effect of TGFβ−1 could be prevented using SB431542, a TGFβ-R inhibitor (expansion index mean ±SD: B-cell + TGFβ−1 + DMSO vs B-cell + TGFβ−1 + SB431542 0.97 ± 0.02 vs. 2.41 ± 0.12 p < 0.0001, Fig. 4C). We also found that expansion and activation of B-cells were correlated with the decrease in checkpoint molecules such as CD22 and CD72 over time, suggesting these B-cells could differentiate into a plasmablast state. However, culturing B-cells with TGFβ−1 prevented the decline of these checkpoint molecules (Fig. 4D–E) which demonstrated that TGFβ−1 could hinder B-cell differentiation. After culturing B-cells with exogenous TGFβ−1, western blots for TRAF6, a molecule recruited downstream of TGFβ-R, confirmed the presence of TGFβ signaling (Fig. 4F). We also showed that TGFβ−1 stimulation increased SHIP1 and SHIP2, critical molecules of checkpoint-mediated ITIM suppression (Fig. 4F).

Because we found that both myeloid and tumor cells produce TGFβ−1 in our murine CT2A model, we generated both Tgfb1 knockout glioma tumor line (CT2A-Tgfb1^KO^ - Tgfb1 gene deletion was validated by western blot and *in-vitro* cell viability was assessed by a mitochondrial function-based assay, cell-titer GLO, Sup Fig. 4A) as well as a transgenic murine line with Tgfb1 KO from myeloid cells (LyzM^cre/WT^-Tgfb1^flox/flox^). Wild-type mice bearing with CT2A-Tgfb1^KO^ tumors had extended survival and increased infiltration of B-cells in the tumor compared to wild-type mice challenged with CT2A-Tgfb1^WT^ tumors (median survival: CT2A-Tgfb1^KO^ vs. CT2A-Tgfb1^WT^ 55 days vs. 29 days, p < 0.05, Fig. 4G–H). Knockout of Tgfb1 from tumor cells also led to increased B-cell proliferation in the brain but no significant increase in regulatory T-cells (Sup Fig. 4B). LyzM^cre/WT^-Tgfb1^flox/flox^ mice with WT CT2A tumors trended towards improved survival compared to control mice with intact Tgfb1 expression by myeloid cells, as well as increased B-cell infiltration into the tumor environment (Fig. 4I–J).

In summary, a screen of B-cell suppression cytokines in the TME environment revealed that myeloid cells across all tumor types produce TGFβ−1, with corresponding high expression of its ligand TGFβ-R2 on tumor B-cells. The data also show that TGFβ−1 directly inhibits B-cell proliferation and downstream activation by maintaining expression of inhibitory checkpoint molecules within the TME.

### Neoadjuvant PD-1 Checkpoint Blockade Therapy Induces B-cell Checkpoint Molecules and Susceptibility to TME Suppression

We evaluated how current immune checkpoint blockade treatment regimens affected the TME B-cell compartment in GBM. Recurrent GBM patients were categorized based on whether they had received neo-adjuvant Pembrolizumab treatment. B-cells in Pembrolizumab treated and untreated were analyzed for checkpoint molecules and suppressive cytokine receptors.

**Figure 5:**
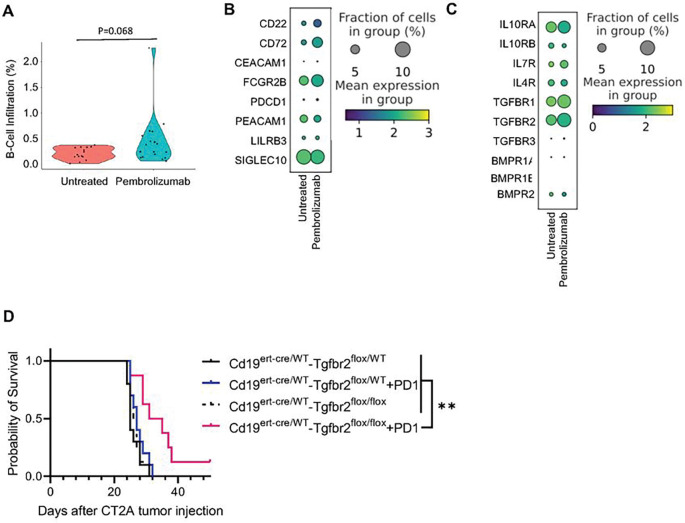
Neoadjuvant PD-1 Blockade in Recurrent GBM patients increase B-cell infiltration but fail to control their suppressed phenotype **(A)** Violin plots of recurrent GBM patients treated with neoadjuvant pembrolizumab (n=18) or without treatment (n=15) showing B-cell infiltration into the tumor. Patients treated with Pembrolizumab trended towards increased B-cell infiltration into the tumor. **(B)** Dot plots of B-cell checkpoint molecule expression in PD-1-treated patients compared to untreated patients. Pembrolizumab increases the expression of B-cell checkpoint molecules, especially *CD22* and *CD72*. **(C)** Dot plots of B-cell suppressive cytokine receptors in PD-1 treated patients compared to untreated patients. Pembrolizumab increases the expression of B-cell suppressive cytokine receptors, especially *TGFBR2* and *IL10R*. **(D)** A transgenic mouse model with an inducible knockout of TGFb-R2 on B-cells (Cd19^ert-cre/WT^-Tgfbr2^flox/flox^) was combined with or without PD-1 blockade and assessed for animal survival compared to Cd19^ert-cre/WT^-Tgfbr2^flox/WT^ controls with or without PD-1 blockade. Cd19^ert-cre/WT^-Tgfbr2^flox/flox^ + PD-1 had significantly improved survival compared to both Cd19^ert-cre/WT^-Tgfbr2^flox/flox^ without PD-1 and Cd19^ert-cre/WT^-Tgfbr2^flox/WT^ + PD-1. ns= p>0.05, * = p<0.05, ** = p<0.01, *** = p<0.001, **** = p<0.0001

The data showed that neo-adjuvant pembrolizumab treatment was correlated with a 2-fold increased B-cell infiltration into the tumor (B-cell infiltration %, No Tx vs pembrolizumab B-cell infiltration %: 0.21 vs 0.44, p = 0.068, Fig. 5A). However, analysis of these intratumoral B-cells revealed that pembrolizumab treatment led to higher expression of *CD22* and *CD72* by B-cells compared to untreated controls (log-fold-change No Tx vs. pembrolizumab: *CD22*, −0.56, p = 0.41; *CD72*, −1.42, p = 0.15), Fig. 5B). Moreover, after pembrolizumab treatment, intratumoral B-cells expressed higher levels of suppressive cytokine receptors, including *TGFBR2* (log-fold-change No Tx vs. pembrolizumab: *TGFBR2*, −0.37, p = 0.26, Fig. 5C).

We hypothesized that pembrolizumab could drive B-cell susceptibility to TME suppression by upregulating TGFβ-R2 on B-cells, leading to increased B-cell checkpoint inhibition. To test this hypothesis, we generated a mouse model with an inducible knockout of *Tgfbr2* in B-cells and treated them with PD-1 blockade (Cd19^ert - cre/WT^-Tgfbr2^flox/flox^). Cd19^ert - cre/WT^-Tgfbr2^flox/flox^ animals had significantly improved response to PD-1 blockade compared to wild type controls and Cd19^ert - cre/WT^-Tgfbr2^flox/flox^ without PD-1 treatment (median survival days: No Tx vs. PD-1 only vs. Cd19^ert - cre/WT^-Tgfbr2^flox/flox^ vs. Cd19^ert - cre/WT^-Tgfbr2^flox/flox^ +PD-1, 25 vs 27 vs 26.5 vs 33, p < 0.01, Fig. 5D).

Our data suggest that while PD-1 blockade is given to unleash anti-tumor function of T-cells, it may hinder anti-tumor B-cell function by increasing B-cell susceptibility to suppressive TGFβ signaling. Thus, targeting both T-cell checkpoints and B-cell suppression may have a synergistic anti-tumor effect.

### Blocking TGFβ and PD-1 Eradicates Tumors in a B-cell Dependent Manner

**Figure 6:**
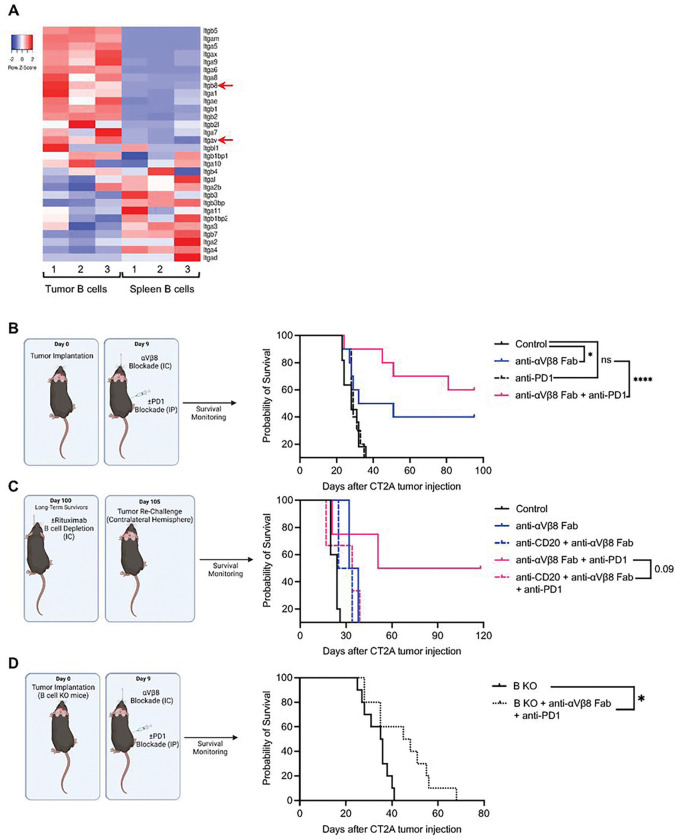
Inhibiting TGFb by blocking αVβ8 integrin synergizes with PD-1 blockade to eradicate tumors in a B-cell-dependent manner **(A)** Bulk RNA sequencing of murine B-cells from CT2A tumors and spleen for various integrin chains. αV and β8 integrin chains are upregulated in tumor B-cells compared to splenic B-cells. **(B)** Survival curves of WT mice challenged with CT2A tumors receiving a combination of αVβ8 integrin blockade and PD-1 checkpoint blockade. Dual therapy with both αVβ8 intregrin blockade and PD-1 checkpoint blockade led to tumor eradication in nearly 60% of mice, significantly outperforming single-agent therapy and untreated controls. **(C)**Survival curves of long-term survivors from (B) that were rechallenged with CT2A tumors on the opposite hemisphere to evaluate immune memory response. Long term survivors in each treatment group were divided into two, with half receiving intracranial CD20 B-cell depleting antibodies prior to tumor rechallenge. Only animals who maintained an intact B-cell compartment and originally received dual αVβ8 integrin blockade and PD-1 checkpoint blockade were able to prevent tumor re-engraphment. **(D)** Survival curves of B-cell knockout mice challenged with CT2A tumors receiving dual αVβ8integrin blockade compared to untreated controls. While the median survival was improved with dual therapy, there was no tumor eradication in any of the groups. ns= p>0.05, * = p<0.05, ** = p<0.01, *** = p<0.001, **** = p<0.0001

While targeting the TGFβ pathway has been attempted in GBM, strategies such as using depletion antibodies or TGFβ receptor blockade has shown minimal clinical efficacy ^64 65^. We targeted the TGFβ pathway in B-cells by blocking the αVβ8 integrin. αVβ8 integrin functions to cleave latency-associated peptide (LAP) to release activated TGFβ ^66^, and is elevated in tumoral B-cells compared to peripheral B-cells (Fig. 6A). To evaluate the therapeutic benefit of αVβ8 integrin blockade and its effects on PD-1 efficacy, wild-type mice were implanted with CT2A tumors and treated with anti-αVβ8 Fab blocking antibodies with or without PD-1 blockade. We use the anti-αVβ8 Fab antibodies to prevent a Fc-mediated B-cell depletion. 200 ug anti-αVβ8 Fab antibodies were given every other day for two weeks. The same regimen was used for PD-1 blockade. While integrin blockade alone provided a significant survival benefit, a combination of anti-αVβ8 Fab and anti-PD-1 antibodies showed a synergistic effect, with nearly 60% of animals having complete tumor eradication (median survival days: No Tx vs. anti-αVβ8 Fab vs. anti-PD-1 vs. anti-αVβ8 Fab + anti-PD-1, 28 vs. 41.5 vs. 29 vs. Undefined, Fig. 6B). Additionally, immune phenotyping of intratumoral B-cells revealed that dual αVβ8 and PD-1 blockade increased B-cell infiltration into the brain tumor, promoted B-cell proliferation, decreased expression of checkpoint inhibitory molecules, and decreased overall population of myeloid cells (Sup Fig. 5A).

To evaluate the effects of B-cells on immunological memory, long-term survivors from αVβ8 blockade and dual αVβ8 + PD-1 blockade groups were split into two groups, with one group receiving intracranial anti-CD20 B-cell depleting antibody and the other group having an intact B-cell compartment. Mice were then re-challenged with CT2A tumors in the contralateral hemisphere. Animals who had initially received dual-αVβ8 + PD-1 blockade and had an intact B-cell compartment were able to mount a memory response, with 50% of mice surviving the tumor re-challenge (median survival days: No Tx rechallenge vs. anti-αVβ8 Fab + anti-PD-1 rechallenge, 24 vs. 84, Fig. 6C). On the other hand, the group with B-cell depletion via anti-CD20 depleting antibody did not show a memory response and extended survival, even with initial dual-αVβ8 + PD-1 blockade treatment (median survival days: anti-αVβ8 Fab + anti-PD-1 rechallenge vs. anti-CD20 + anti-αVβ8 Fab + anti-PD-1 rechallenge, 84.5 vs. 34, Fig. 6C). To further evaluate the role of B-cells on the therapeutic benefit, the dual-αVβ8 + PD-1 blockade was given to B-cell knockout mice. Whereas wild-type mice saw significant survival benefit and tumor clearance from the combinational treatment with dual integrin and PD-1 blockade therapy (Fig. 6B), B-cell knockout mice only showed minor survival benefit and no tumor clearance (median survival days: B-cell KO + No Tx vs B-cell KO + anti-αVβ8 Fab + anti-PD-1, 35.5 vs. 46.5, p < 0.05, Fig. 6D).

These data show that B-cells play an essential role in the anti-tumor effect of PD-1 blockade. Mitigating B-cell immunosuppression induced by PD-1 blockade by combining checkpoint inhibition with αVβ8 integrin blockade significantly enhances anti-tumor immunity.

### TME Suppresses B-cell Plasmablast Differentiation and Antibody Production

**Figure 7:**
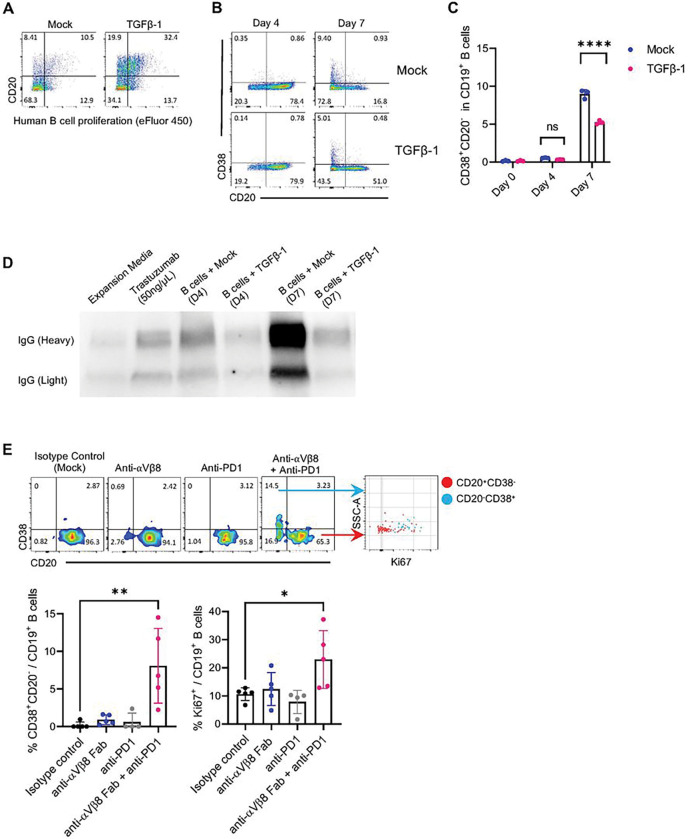
TGFb-1 and TME Suppress B-cell Plasmablast Differentiation and Antibody Production **(A)** Representative flow cytometry plots of B-cells activated with B-cell expansion cocktail showing association between B-cell proliferation (loss of eFluor 450 proliferation dye) and decrease in CD20 expression. TGFb-1 inhibits both proliferation and loss of CD20. **(B-C)** Representative flow cytometry plots and bar plots of B-cells activated with B-cell expansion cocktail showing TGFb-1 inhibits B-cell differentiation into plasmablasts (CD38+CD20−). **(D)** Western blots for IgG heavy and light chains in B-cell culture media. The same number of B-cells (0.5×10^6^ ) were cultured in antibody-free media and activated with B-cell expansion cocktail. TGFb-1 was added to the experimental group. The supernatant was sampled at different time points for analysis of IgG antibodies. **(E)** Representative flow cytometry plots and bar graphs of intratumoral B-cell plasmablast differentiation (CD38+CD20−) in tumor-bearing mice after no treatment, mono treatment with αVβ8 blockade or PD-1 blockade, or dual αVβ8 and PD-1 blockade. Plasmablast and total B-cell proliferation were assessed with Ki67 expression. ns= p>0.05, * = p<0.05, ** = p<0.01, *** = p<0.001, **** = p<0.0001

Having shown that mitigating B-cell suppression using αVβ8 integrin blockade can increase the efficacy of checkpoint blockade and promote tumor clearance, we then evaluated how the combinational therapy affected the function of intratumoral B-cells. Using our *in-vitro* B-cell culture system, we studied the effects of TGFβ−1 on human B-cell differentiation into plasmablasts (CD38^+^CD20^−^) ^67 68^. First, we confirmed that B-cells in our expansion media could differentiate into plasmablasts, as B-cell proliferation was associated with a loss of CD20, but TGFβ−1 prevented both proliferation and CD20 loss (Fig. 7A). Additionally, analysis of B-cells for plasmablast phenotype markers (CD38^+^CD20^−^) showed increases in plasmablast differentiation over seven days of culture with the B-cell expansion media. Plasmablast differentiation was significantly suppressed by TGFβ−1 (Fig. 7B–C). Moreover, western blots for IgG in our B-cell culture media showed that B-cells cultured with TGFβ produced less IgG (Fig. 7D).

To evaluate how integrin and checkpoint blockade could affect B-cell differentiation into plasmablasts *in-vivo*, CT2A tumor-bearing mice were treated with αVβ8 or PD-1 blockade monotherapy or a combination of αVβ8 and PD-1 blockade every other day for two weeks. Two weeks after tumor implantation, B-cells were isolated from the brain and analyzed for plasmablast differentiation. As we saw in our single-cell GBM analysis of B-cell phenotype in the tumor, CT2A tumor-bearing mice without any treatment showed minimal amounts of intratumoral plasmablasts (Fig. 7E). While treatment with only αVβ8 blockade or only PD-1 blockade did not show a significant increase of plasmablasts in the brain, dual αVβ8 and PD-1 blockade led to a substantial increase of total plasmablasts (CD38^+^CD20^−^) and proliferating B-cells (%CD38^+^CD20^−^CD19^+^ plasmablasts mean ± SD: isotype control treatment vs. anti-αVβ8 + anti-PD-1 treatment, 0.1920 ± 0.384 vs. 8.076 ± 4.442, p < 0.01) (%Ki67^+^CD19^+^ proliferating B-cells mean ± SD: isotype control treatment vs. anti-αVβ8 + anti-PD-1 treatment, 10.61 ± 2.051 vs. 22.96 ± 9.175, p < 0.05, Fig. 7E).

Thus, our results suggest that combination treatment with αVβ8 and PD-1 blockade can rescue B-cell function by promoting B-cell proliferation and plasmablast differentiation even in the suppressive TME.

#### Blocking B-cell Suppression Promotes T-cell Expansion and Function.

**Figure 8:**
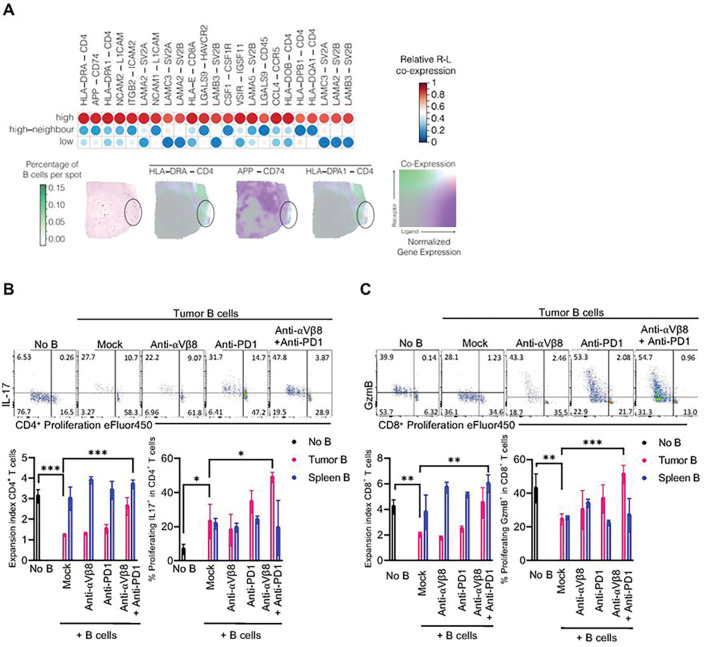
Blocking B-cell Suppression Promotes T-cell Expansion and Function **(A)** Heatmap of receptor-ligand interaction abundance across B-cell-enriched spots. At the bottom, a representative example of receptor-ligand interactions and B-cell accumulation is shown. **(B)** CD8+ T-cell suppression assay with B-cells isolated from tumors that underwent no treatment, mono therapy with PD-1 or αVβ8 blockade, or dual PD-1 and αVβ8 blockade. T-cell proliferation was measured via dilution of proliferation dye eFluor 450, and effector function was measured by IL17 expression. **(C)**CD8+ T-cell suppression assay with B-cells isolated from tumors that underwent no treatment, mono therapy with PD-1 or αVβ8 blockade, or dual PD-1 and αVβ8 blockade. T-cell proliferation was measured via dilution of proliferation dye eFluor 450, and effector function was measured by GzmB expression. ns= p>0.05, * = p<0.05, ** = p<0.01, *** = p<0.001, **** = p<0.0001

The role of B-cells as antigen-presenting cells capable of activating CD8 and CD4 T-cells is another critical anti-tumor function beyond antibody generation ^20 13^. Spatial transcriptomic analysis of patient GBM samples revealed that in areas of high B-cell infiltration, interactions between HLA and CD4 and CD8 were highly enriched (Fig. 8A). Under normal conditions, intratumoral B-cells in GBM are immunosuppressive due to the conversion by myeloid cells into regulatory B-cells ^12^.

We performed a T-cell suppression assay where activated T-cells were cultured with B-cells isolated from murine CT2A tumors. CD4^+^ and CD8^+^ T-cells cultured with B-cells isolated from untreated tumors, or B-cells isolated from tumors treated with single-agent αVβ8 blockade or PD-1 blockade, showed significant suppression as measured by proliferation and IL17 or granzyme B expression (CD4^+^ T-cell expansion index mean ± SD: No B-cells vs. Tumor B-cells + mock treatment, 3.13 ± 0.26 vs. 1.23 ± 0.04, p < 0.001, Fig. 8B). (CD8^+^ T-cell expansion index mean ± SD: No B-cells vs. Tumor B-cells + mock treatment, 4.18 ± 0.46 vs. 2.03 ± 0.17, p < 0.01, Fig. 8C).

However, CD4 T-cells cultured with intratumoral B-cells isolated from animals treated with dual αVβ8 and PD-1 blockade were no longer suppressed, showing significantly increased proliferation and IL17 expression (T-cell expansion index mean ± SD: Tumor B-cells + mock treatment vs. Tumor B-cells + anti-αVβ8 + anti-PD-1, 1.27 ± 0.04 vs. 1.96 ± 0.12, p < 0.01)(T-cell %proliferating IL17^+^ mean ± SD: Tumor B-cells + mock treatment vs. Tumor B-cells + anti-αVβ8 + anti-PD-1, 22.90 ± 8.32 vs. 48.73 ± 2.54, p < 0.05, Fig. 8B). The same T-cell suppression assay was performed with intratumoral B-cells and CD8 T-cells, showing the similar effects that we observed in CD4 T-cells with increased CD8 T-cell proliferation and granzyme B expression in T-cells mix-cultured with B-cells derived from mice treated with dual αVβ8 and PD-1 blockade (T-cell expansion index mean ± SD: Tumor B-cells + mock treatment vs. Tumor B-cells + anti-αVβ8 + anti-PD-1, 2.03 ± 0.17 vs. 4.50 ± 0.98, p < 0.05)(T-cell %proliferating GzmB + mean ± SD: Tumor B-cells + mock treatment vs. Tumor B-cells + anti-αVβ8 + anti-PD-1, 24.33 ± 2.67 vs. 51.13 ± 4.54, p < 0.01, Fig. 8C).

The data suggest that mitigating B-cell suppression in the TME can enhance the function of T-cells by leveraging the antigen presentation function of B-cells. Our study provides a strategy to combine T-cell checkpoint blockade and B-cell TGFβ pathway blockade to prompt a synergistic anti-tumor efficacy.

## Discussion

Our study highlights a mechanism of brain tumor-mediated B-cell suppression that can be leveraged to augment the effects of immunotherapy. We quantified B-cell infiltration in GBM using spatial transcriptomics and characterized them as antigen-experienced but functionally inhibited via ITIM checkpoint molecules, a shared phenotype across GBM and MBM. Analysis across different types of human tumors suggests that a brain-intrinsic property prevents complete differentiation of B-cells into plasmablasts, as GBM B-cells had the fewest proportion of plasmablasts, and MBM had fewer plasmablasts compared to ECM. Furthermore, GBM B-cells had decreased checkpoint molecule expression compared to PBMC B-cells but still maintained expression of CD72 and CD22, suggesting that B-cells are activated upon recruitment to the brain, but the TME prevents complete transition into plasmablasts. While GBM and MBM share a lack of plasmablasts, their inhibitory checkpoint molecule expression profile had subtle differences, indicating that GBM and MBM still have unique tumor properties and TMEs.

As current development and study of immunotherapy focus primarily on T-cells, very little are known about how checkpoint inhibitors, directly and indirectly, affect other cell types. We showed that neoadjuvant Pembrolizumab treatment in recurrent GBMs sensitized B-cells to immune suppression by increasing the expression of TGFβ-R2 and inhibitory checkpoint molecules. B-cells are known to express PD-1, and thus it is possible that pembrolizumab directly affects B-cells. Because checkpoint blockade significantly increases T-cell activity, B-cell suppression can also be viewed as a potential mechanism of homeostasis to prevent immune toxicities.

Tumor-associated myeloid cells are highly prevalent in the GBM TME, and their role in immunosuppression has been well studied. The suppressive role of TAMs often focuses on the immunosuppression of T-cells, but our spatial transcriptomic data in GBM samples highlight the strong spatial correlation between myeloid cells and B-cells. Our previous work highlighted the role of TAM-derived microvesicles that deliver PD-L1 to the surface of B-cells, forming regulatory B-cells that suppress T-cell functions^12^. Here, we also highlight a new mechanism of B-cell suppression via soluble cytokines, TGFβ−1 in particular, in multiple brain tumor models. However, between MBM and ECM, different subtypes of myeloid cells play more important roles in TGFβ-mediated B-cell suppression, further suggesting brain-intrinsic factors that affect myeloid cell function.

While previous clinical trials have tested both soluble TGFβ blockers and TGFβ receptor inhibitors, significant therapeutic benefits have yet to be seen ^64,65^. TGFβ is a well-studied cytokine with many critical pro and anti-inflammatory functions, such as maintenance of naïve lymphocyte populations ^69^; thus, nonspecific inhibition of TGFβ may induce undesired immune suppression outside of the TME, weakening anti-tumor effects. Other studies have shown that direct targeting of TGFβ on specific cells by inhibiting the αV integrin on NK cells ^70^ or αVβ8 integrin on regulatory T-cells ^66^ can augment their cytotoxic abilities or reduce their immunosuppression.

Targeted blockade of TGFβ signaling by inhibiting αVβ8 integrin on B-cells, in combination with PD-1 checkpoint blockade, led to significant B-cell dependent tumor eradication and immune memory, restoring plasmablast differentiation and antigen presentation function. PD-1 therapy enhances T-cell immunity and increases humoral B-cell infiltration; however, it fails to control B-cell suppressive phenotype. Combining T-cell checkpoint blockade and indirect B-cell checkpoint blockade via integrin blockade allowed for more dramatic TME immune remodeling and anti-tumor effects. αVβ8 integrin is also expressed on other cell types such as CNS cells and the tumor ^71 72^ - thus αVβ8 integrin blockade can potentially have direct anti- TGFβ effects on tumor cells as well.

The role of B-cells in the TME can be broadly categorized in 3 main categories: antibody production ^40 73^, antigen presentation ^21^, and cytokine secretion ^74^. We demonstrate an increase in plasmablast differentiation in the TME after dual αVβ8 integrin and PD-1 blockade, and future work will be done to characterize potential antibody reactivity. While our study focused on IgG production, previous studies have shown that TGFβ-R2 deletion in murine B-cells leads to a complete absence of IgA in the serum ^75^ - thus further work can be done to study the effects of our dual αVβ8 integrin and PD-1 blockade on Ig class switching.

We also identified increased spatial co-localization and co-expression of HLA and both CD4 and CD8 molecules in B-cell-rich zones, suggesting formation of potential germinal center-like structures with T-cell activation. Moreover, cell-cell interactome analysis based on spatial transcriptomic identified increased interaction between B-cells and NK cells. Such B-cell-NK interactions could suggest antibody dependent cytotoxicity or B-cell cytokine production ^76^. The reverse interaction can also be possible, where NK cells in the TME can regulate or activate B-cells through interferon signaling ^77^.

Overall, our study proposes the importance of leveraging B-cell immunity to target both GBM and other brain tumors. Blockade of αVβ8 integrin promotes B-cell effector function in a B-cell-specific manner, making its clinical translation highly feasible. Future directions of this study should revolve around utilizing insights into TME immunosuppression to develop a new generation of therapies with potent cytotoxicity and mechanisms of protection against the TME integrated into their designs.

## Materials And Methods

### Human Samples

The Nervous System Tumor Bank at Northwestern University was used for the collection of all human samples. Approval for collection was granted under the institutional review board protocol number STU00202003 and the study was conducted following the U.S. Common Rule of ethical standards. All patients signed written consent forms. Collected samples included tumor, peripheral blood, and frozen tissue from GBM patients with at least 50% tumor cellularity, as determined by neuropathologist review of H&E sections.

### Mice

C57BL/6, B-cell Knockout (μMT), Nur77-GFP, CD19^Cre^, LyzM^Cre^, Tgfb1^flox^, and Tgfbr2^flox^ were all purchased from The Jackson Laboratory and bred for use in experiments. Studies were initiated when the mice were 6–8 weeks old. Approval for all animal experimental protocols were approved by the Institutional Animal Care and Use Committee at Northwestern University under the protocol number ISO16696. All animals were housed at the Center for Comparative Medicine at Northwestern University in a dedicated pathogen-free animal facility with 12-hour light/12-hour dark cycles and ad libitum access to food and water.

### Cell Lines

CT2A cells were obtained from Millipore (Sigma Aldrich). Cells were cultured in Dulbecco’s modified Eagle’s medium (Corning) supplemented with 10% fetal bovine serum (FBS; Hyclone), 100 U/ml penicillin (Corning), and 100 mg/ml streptomycin (Corning) and incubated at 37°C in 5% CO_2_. Every 2 months cell lines were tested for *Mycoplasma* contamination using the Universal Mycoplasma Detection Kit (ATCC 30–1012K).

### Generation of single gene knockout of TGFβ−1 in CT2A glioma cells

Single gene knockout clones were generated in lentiCRISPRv2 (one vector system). The vector backbone was purchased from Addgene (lentiCRISPR v2 was a gift from Feng Zhang (Addgene plasmid # 52961; http://n2t.net/addgene:52961; RRID:Addgene_52961) ^78^. The protocol for guide cloning and generation of the virus was as described in Sanjana *et al*.^78^. The guide sequence for mouse TGFβ−1 KO is “CACCGTTGACGTCACTGGAGTTGTA” and non-targeting control (NTC) is “CACCAATATTTGGCTCGGCTGCGC”. The TGFβ−1 KO and control clones were selected using puromycin from Sigma (2ug/ml) in CT2A mouse glioma cell line. The TGFβ−1 KO was confirmed using western blotting (TGFβ−1 antibody from Abcam [EPR21143] (ab215715)).

### Cell Titer Glo

CT2A-Vector control and CT2A-Tgfb1^KO^ cells were seeded in a white 96-well flat bottom plate at a density of 1×103 cells/well in 100ul complete DMEM. Twelve hours after seeding cells were checked for adherence and a “Day 0” Cell-Titer Glo reading was done. The Cell-Titer Glo assay (Promega) was done by adding 100uL of a 1:3 dilution of Cell Titer-Glo Reagent: PBS directly to the 100uL of cells in cDMEM. The plate was incubated on a plate shaker with constant shaking for 10 minutes. Following incubation plate was placed in a BioTek Cytation5 plate reader and luminescence signal was measured with the following settings:

Luminescence, Read Type: Endpoint/Kinetic, Optics Type: Luminescence Fiber, Gain: 135, Integration Time: 0:01.00 (MM:SS.ss), Read Height: 1.00mm.

Readings were taken every 24 hours for the next 5 days

### Intracranial Tumor Injections

Each mouse was injected with 1×10^5^ tumor cells in a total volume of 2.5ml of PBS. Mice were anesthetized with ketamine (100 mg/kg) and xyalizine (10 mg/kg) via intraperitoneal injection. After shaving of surgical site and disinfection with povidone-iodine and 70% ethanol, an incision was made at the midline for access to the skull. A 1 mm-diameter burr hole was drilled 2mm posterior to coronal suture and 2 mm lateral to the sagittal suture. Injections were performed using a Hamilton syringe fitted with a 26-guage blunt needle at a depth of 3.5mm. The injection site was then sutured closed.

### Intracranial Cannula Implantation

Mice were anesthetized, and a skin incision ~10 mm in length was made over the middle frontal to the parietal bone to expose the surface of the skull. A 26-gauge sterile guide cannula for mice (Plastics One) was installed into the mouse brain at 2-mm depth through the burr hole generated during tumor implantation, as described above. Tissue glue was applied around the burr hole to secure the protrusion of the cannula for long-term stable positioning. The scalp was closed with surgical glue around the implantation site. A protection dummy cannula was used to secure the protrusion end during the postop recovery and following observation period.

### Spatial multi-omics Analysis

For spatial data analysis, we acquired the spatially resolved RNA-seq datasets using the *SPATAData* package^50^ (https://github.com/theMILOlab/SPATAData). Data processing and visualization were performed by the SPATA2 package (https://github.com/theMILOlab/SPATA2) ^50^.

### Spatial weighted Correlation analysis

For spatial weighted correlation analysis, we used the SPATAwrappers package and the *runSpatialRegression* function. Spatially correlation analysis was performed by either a spatial Lag model or a Canonical Correlation Analysis (CCA).

### Cell Type Deconvolution

Cell type deconvolution of each spot was performed by RobusT-cell Type Decomposition (RCTD) a well-validated toolbox^79^. The deconvolution was performed by the SPATAwrappers (https://github.com/heilandd-/SPATAwrappers) package using the function *runRCTD*. Visualization of surface plots or correlation analysis was performed by the SPATA2 toolbox.

### Spatial Receptor Ligand Interactions

Recepor-ligand interaction analysis was performed by the NFCN2 package (https://github.com/heilandd/NFCN2). First we identified spots with B-cell accumulation (as explained before), neighborhood spots (with a distance of 110 μm) and non B-cell enriched spots. We next quantified the co expression of receptor and ligand pairs using the NFCN2 databases. Co expression visualization was performed by the SPATAwrappers package using the *plotColorOverlap* function.

### Single-cell and single-nuclei RNA Sequencing and Analysis

For human GBM samples, public single-cell RNAseq of GBM brains, as well as RNAseq from two paired tumor and PBMC patient samples, were used for the analysis. Single-cell preparation was done using the adult brain dissociation kit for mouse and rat (Miltenyi Biotec). After ensuring cell viability of greater than 70%, cells were given to the RNA sequencing core at Northwestern University for further processing and sequencing.

For murine single-cell transcriptomic analysis, intracranial tumors from CT2A-bearing C57BL/6 mice were dissected from the brains of mice 14 days after CT-2A tumor implantation. Three tumors were pooled for this analysis. Single-cell preparation was done using the adult brain dissociation kit for mouse and rat (Miltenyi Biotec). After ensuring cell viability of greater than 70%, cells were given to the RNA sequencing core at Northwestern University for further processing and sequencing.

Single-cell transcriptomic dataset for untreated recurrent GBM and neo-adjuvant treated GBM samples (UCLA dataset) were acquired from Cloughesy et al. ^80^ and Lee et al. ^81^

Single-nuclei RNA-seq data of human melanoma brain and extracranial metastases was obtained from Biermann, Melms et al. ^82^ (GEO: GSE185386). UMAPs and dot plots were generated as described in Biermann, Melms et al. ^82^ using the Seurat R-package ^83^ (v4.1.0, https://github.com/satijalab/seurat/).

To visualize the single-cell RNA-seq results, the normalized gene barcode matrix was used to compute a neighborhood graph of cells, then Uniform Manifold Approximation and Projection (UMAP) was performed with default parameters. The whole pipeline was implemented using Scanpy ^84^. Cell type annotation was performed using R package SingleR ^85^. Differential expression analysis was performed by Wilcoxon rank-sum test between groups. GSEA of selected pathways was performed using the Python package GSEApy (https://gseapy.readthedocs.io/en/latest/index.html).

### Bulk RNA Sequencing and Analysis

14 days after implantation, tumors were isolated from 10 mice for each *n* included in this study and enriched for CD19 using biotinylated CD19 antibody (Biolegend) and anti-biotin microbeads (Miltenyi Biotec). RNA from these samples was isolated using TRIzol (Thermo Fisher Scientific) to purify. Chloroform (0.2ml) was added to the TRIzol samples. The top layer containing the RNA was precipitated with 70% isopropanol. The resulting pellets were dried, resuspended in sterile water, and sent to Novogene for further processing and analysis. Novogene assessed the RNA for quality and provided all data as total counts and fragments per kilobase per million reads (fpkm).

### Multiplex immunofluorescence staining

Sections of 5μm thickness were obtained from FFPE embedded tumor tissue. Deparaffinization of the slides was achieved using xylene followed by rehydration in histological grade ethanol and fixed with 3% hydrogen peroxide in methanol before heat-induced epitope retrieval using BOND epitope retrieval solution (pH6) or pH9 EDTA buffer for 20 minutes. 3,3’-diaminobenzidine chromogen staining was initially performed to determine the optimal concentrations of each antibody in human GBM tissues. Primary antibodies were diluted with 1x Opal Antibody diluent/block solution and were used in the following order coupled with the indicated Opal dyes: 1) TMEM119 (cat HPA051870, Sigma-Aldrich, 1:250 dilution) with Opal 520 (1:100 dilution), 2) CD163 (cat ab213612, clone EPR19518, Abcam, 1:600 dilution) with Opal 570 (1:800 dilution), 3) SOX2 (cat ab92494, clone EPR3131, Abcam, 1:5000 dilution) with Opal 620 (1:150 dilution), 4) TGFβ (cat ab92486, Abcam, 1:100 dilution) with Opal 690 (1:200 dilution), 5) CD20cy (cat M075501–2, clone L26, Agilent Dako, 1:400 dilution) with Opal 540 (1: 200 dilution), 6) CD8 (cat CD8–4B11-L-CE, clone 4B11, Leica Biosystems, RTU) with Opal 650 (1:100 dilution). Multiplex staining was performed in multiple cycles involving a heat-induced epitope retrieval step, protein blocking, epitope labeling, and signal amplification. Once all markers were stained, spectral DAPI was used to counterstain the slides and were mounted using Prolong Diamond Antifade Mountant.

### Imaging and analysis of multispectral images

Multispectral imaging (MSI) was performed using the Vectra 3 Automated Quantitative Pathology Imaging System from Akoya Biosciences. First, whole slide images were acquired after auto-adjusting focus and signal intensity. Then, MSI was acquired from the tumor regions delineated by a certified neuropathologist at 20x of the original magnification. For analysis of MSI, we created a spectral library for all Opal dyes to subject acquired multispectral images to spectral unmixing that enabled the identification and separation of weakly expressing and overlapping signals from background to visualize the signal of each marker (SOX2, CD20cy, TMEM119, CD163, TFG-b, CD8, DAPI) in inForm Tissue Finder software (inForm 2.6, Akoya Biosciences). Using InForm, the adaptive cell segmentation feature was used to identify the analyzed cells’ nuclei and determine each cell’s nuclear and cytoplasmic compartments. A machine-learning algorithm within inForm was used in which cells were automatically assigned to a specific phenotype (SOX2+, TMEM119+, CD163+, CD20+, CD8+, TFG-b+). Batch analysis was used to analyze all tumor samples under the same segmentation and phenotype settings. The processing and analysis of images from all tumor samples were exported to cell segmentation tables. Exported files from inForm were processed in R using R packages Phenoptr and PhenoptrReports to merge and create consolidated single files for each tumor sample. Consolidated files had triple cell phenotypes as outputs that we employed for further quantification and spatial analyses using the Phenoptr R addin.

#### Quantification of cell types and spatial analysis of GBM samples.

Merged and consolidated files were analyzed using Phenoptr to quantify the cell density of SOX2 + TGFβ−1+, TMEM119 + TGFβ−1+, CD163 + TGFβ−1+, TMEM119 + CD163− TGFβ−1+, and CD163 + TMEM119− TGFβ−1+, CD20+, CD8+. For the spatial analysis, mean cell counts within a specified radius of 15 mm from a given cell type to another were calculated using Phenoptr as an R addin. Then, mean distances between the nearest neighbors were calculated from particular cell types to other cell types. The spatial map viewer addin within R allowed the visualization of the nearesT-cell neighbor between selected phenotypes in a single field. Cartoons were created using Adobe Illustrator version 22.1.0.

### Isolation of GBM infiltrating Immune cells and PBMCs

Freshly resected GBM tumor samples were collected and transported in complete RPMI media (RPMI + 10% heat-inactivated FBS, 10μM HEPES-sodium pyruvate, 1 mM sodium pyruvate, 0.01% 2-mercaptoethanol, 2-mM L-glutamine, penicillin (100 U/mL), and streptomycin (100ug/mL); all reagents from Thermo Fisher). Samples were manually diced with a razor blade and enzymatically digested using the Adult Brain Digestion Kit (Miltenyi Biotec) according to manufacturer instructions.

Peripheral blood samples from GBM patients were collected in EDTA tubes. Peripheral blood mononuclear cells (PBMC) were isolated using the Ficoll (GE Healthcare) gradient. Tumor cells and PBMCs were immediately put in complete RPMI media after isolation.

### In-vitro B-cell Culturing and Phenotypic Analysis

Human B-cells were isolated from healthy donor PBMCs using the Easysep B-cell isolation kit (Stemcell Technologies). Isolated B-cells were cultured in ImmunoCult-XF T-cell Expansion Medium supplemented with ImmunoCult-ACF Human B-cell Expansion Supplement (Stemcell Technologies) for up to 7 days. For cells cultured with TGFβ−1, recombinant human TGFβ−1 (Peprotech, 10ng/mL) was added every day for the duration of the culture. For cells also cultured with SB431542 TGFβ receptor inhibitor, SB431542 (10uM) was 30 minutes before the addition of TGFβ−1.

Cells were labeled with Fixable Cell Proliferation Dye eFluor-450 (eBioscience) or stained with antibodies (all from Biolegend) CD19 PerCP-Cy5.5, CD22 Pacific Blue, CD72 APC, CD38 Alexa-Fluor700, and CD20 Pe-Cy7. In all experiments, cells were first incubated in Fc-Block (Biolegend), and dead cells and debris were excluded from analysis using Fixable Viability Dye eFluor780 (eBioscience). Cells were acquired by BD Symphony and analyzed using the FlowJo software.

### Western Blots

Cells were removed from the medium, washed once with ice-cold PBS, and lysed in ice -cold mammalian protein extraction reagent (M-PER; Thermo Scientific, Rockford, IL, USA) supplemented with protease and phosphatase inhibitor cocktail (PPI; Thermo Scientific, Rockford, IL, USA). Cell lysates were sonicated in a water bath in 3×30-second increments, with 30 seconds of rest between each sonication. The resulting lysates were centrifuged at 14,800 rpm for 10 min at 4°C. Following supernatant collection, the protein concentration for each western blot sample was determined via BSA assay (Thermo Scientific, Rockford, IL, USA). Lysate concentrations were normalized,, and 4x laemmli sodium dodecyl sulfate buffer (SDS sample buffer; Boston BioProducts, Boston, MA, USA) was added. Samples were boiled at 95°C for 10 minutes in preparation for gel loading. Samples were then loaded and run through 4–15% SDS-polyacrylamide gels (BioRad, Hercules, CA, USA) at 45V initially and then 100V until completion. Proteins were transferred onto 0.2μm polyvinylidene difluoride (PVDF) membranes (Millipore, Darmstadt, Germany) at 14V constant in a Trans-Blot semi-dry transfer machine. Following the transfer, the membranes were rinsed in distilled water briefly and transferred to a blocking solution(5% powdered milk made in TBS-T) for one hour at room temperature. Following block, membranes were washed in TBS-T 3×10 minutes and incubated in primary antibody solution overnight at 4°C. Primary solutions were made 1:1000 in 5% bovine serum albumin solution supplemented with sodium azide 0.02% (w/v) (Cell Signaling, TRAF6 (D21G3) Rabbit mAb #8028, SHIP1 (P290) Antibody #2726, SHIP2 Antibody #2730). The next morning, membranes were washed in TBS-T 3×10 minutes and incubated at room temp in secondary antibody (Cell Signaling, Anti-rabbit IgG, HRP-linked Antibody #7074) diluted 1:4000 in 5% milk for 1 hour. Following secondary another 3×10 minutes wash cycle in TBS-T, membranes were coated with an enhanced chemiluminescence solution (ECL; Clarity ECL, BioRad), and images were developed in a developer machine (BioRad, Hercules, CA, USA).

### αVβ8 Blockade and PD-1 Blockade

CT2A-bearing mice were treated with 200ug/mouse αVβ8 recombinant antibody Fab fragment (clone C6D4, Creative Biolabs) intracranially via cannula every other day for two weeks. If the mouse received PD-1 blockade, 200ug/mouse PD-1 antibody (clone RMP-14, BioXcell) was delivered every other day via intraperitoneal injection.

### Isolation of Murine Intratumoral B-cells

Ex-vivo analysis of tumoral and peripheral immune cells was performed 14 days after tumor injection. Animals were euthanized and perfused with PBS before organs were harvested. Brains were mechanically dissociated using a tissue homogenizer (Potter-Elvehjem PTFE pestle) in HBSS. Cell clumps were removed using a 70 μm cell strainer (Thermo Fisher). Red blood cells, myelin, and debris were removed by a 30/70 Percoll (GE Healthcare) gradient separation (30min, 1000× g at room temperature). The immune cell layer was collected and used for downstream analysis.

### Flow Cytometry and Immunophenotype Analysis

Immunophenotype analysis of immune cells from tumor-bearing mice was performed at time points defined in the results section. After collecting single-cell suspensions, cells were counted and washed with staining buffer (5% bovine serum albumin, 0.001% sodium azide in PBS). Cells were incubated with 1uL Fc receptor blocking Ab (anti-CD16/32, clone 93, Biolegend) per million cells in 100uL staining buffer for 5 minutes at room temperature. For surface staining, cells were incubated with 1uL Ab per million cells for 30 minutes at 4°C. Cells were stained with Fixable Viability Dye eFluor 780 (eBioscience, Thermo Fisher) for 30 minutes at 4°C. Cells were washed twice with staining buffer. Cells were fixed and permeabilized for intracellular staining using the eBioscience Foxp3/Transcription Factor Staining Buffer Set (Invitrogen, Thermo Fisher) for 90 minutes at room temperature. Cells were washed twice with the permeabilization buffer included with the kit and incubated with 1uL Ab for 1 hour at 4°C. Data was acquired with BD FACS Symphony analyzer and analyzed with FlowJo software. Dead cells and debris were excluded using the Live/Dead staining (Viability Dye eFluor 780). B-cells were identified as CD45 + CD11b-CD19 + cells.

### T-cell suppression assay

CD8 + T-cells were isolated from spleens of naïve mice using EasySep Mouse CD8 + T-cell Isolation Kit (StemCell Technologies). Cells were labeled with the eBioscience cell proliferation dye eFluor 450 (Thermo Fisher). B-cells were isolated from tumor bearing brains using CD45 and CD19 antibody selection (Biolegend). To test B-cell ability to suppress activated CD8 + and CD4 + T-cell proliferation, cells were mixed at a 1:1 B:T ratio. T-cell activation was assessed using the anti-CD3/CD28 T-cell activating beads used at 1:3 beads:T-cell ratio (Invitrogen, Thermo Fisher) plus IL2 (50U/mL, Peptrotech) for 3 days in complete RPMI. T-cell proliferation (eFluor450 dye dilution) and effector T-cell factors such as granzyme B and IL17 were analyzed by flow cytometry.

### Data and Code Availability

UCLA GBM scRNAseq data was downloaded from GEO GSE154795. We also used another public dataset from GEO GSE182109. All the custom code will be made available upon request.

### Statistical Analysis

Data are shown as mean ± standard deviation for continuous variables and numbers for categorical variables. Differences between 2 groups were analyzed using Student’s t test. Multiple groups were analyzed using one-way ANOVA with a post hoc Tukey’s multiple comparisons test. Survival curves were analyzed and generated using the Kaplan-Meier method and compared by log-rank test. Categorical variables were analyzed using Fisher’s exact tests or X^2^ tests as appropriate. All tests are two-sided with p values or Benjamini-Hochberg adjusted false discovery rates of < 0.05 were considered significant. Statistical analyses were performed using GraphPad Prism 9.4.1. ns = p > 0.05, * = p < 0.05, ** = p < 0.01, *** = p < 0.001, **** = p < 0.0001.
